# Anaerobic digestion of pig manure supernatant at high ammonia concentrations characterized by high abundances of *Methanosaeta* and non-euryarchaeotal archaea

**DOI:** 10.1038/s41598-017-14527-1

**Published:** 2017-11-08

**Authors:** Anna Synnøve Røstad Nordgård, Wenche Hennie Bergland, Olav Vadstein, Vladimir Mironov, Rune Bakke, Kjetill Østgaard, Ingrid Bakke

**Affiliations:** 1 Department of Biotechnology and Food Science, Norwegian University of Science and Technology (NTNU), Sem Sælands vei 6/8, 7491 Trondheim, Norway; 2 Department of Process, Energy and Environmental Technology, University College of Southeast Norway (USN), Kjølnes ring 56, 3918 Porsgrunn, Norway

## Abstract

We examined the effect of ammonium and temperature on methane production in high rate upflow anaerobic sludge bed reactors treating pig manure supernatant. We operated four reactors at two ammonium concentrations (‘low’ at 1.9, ‘high’ at 3.7 g L^−1^, termed LA and HA reactors, respectively) and at variable temperatures over 358 days. Archaeal and bacterial communities were characterized by Illumina sequencing of 16S rRNA amplicons. Ammonium was a major selective factor for bacterial and archaeal community structure. After ~200 days of adaptation to high ammonium levels, acetate and propionate removal and methane production improved substantially in HA reactors. Aceticlastic *Methanosaeta* was abundant and positively correlated to methane yield in the HA reactors, whereas *Methanosarcina* was more abundant in LA reactors. Furthermore, a group of monophyletic OTUs that was related to Thaumarchaeota in phylogenetic analysis was highly abundant in the archaeal communities, particularly in the HA reactors. The most abundant bacterial OTU in LA reactors, representing *Syntrophomonadaceae*, was also positively correlated to methane yield in the HA reactors, indicating its importance in methane production under ammonia stress. In conclusion, efficient methane production, involving aceticlastic methanogenesis by *Methanosaeta* took place in the reactors at free ammonia concentrations as high as 1 g L^−1^.

## Introduction

On-farm anaerobic digestion has been suggested as an attractive option for manure treatment, as it reduces greenhouse gas emissions from the agricultural sector, it produces high quality biogas and reduces other environmental impacts such as water pollution and odour emissions^1^. We recently demonstrated that high rate UASB reactors, with hydraulic retention time as low as 2 hours, can be used for anaerobic digestion of supernatant rich in particles from cow and pig manure, and that the process is well suited for on-farm manure treatment^2,3^. Pig manure is ammonia rich. This is a potential problem in anaerobic treatment, as high ammonia concentrations inhibit the methanogenesis and result in poor biogas yields^4,5^, even though adaptation of the anaerobic microbial community has been shown to increase the tolerance to ammonia^6,7^. Free ammonia nitrogen (FAN, NH_3_) is the component causing the inhibition, and the FAN concentration depends mainly on the total ammonia concentration, pH, and temperature^6^. Acetate is a major intermediate in methanogenesis, and may be converted to methane through two distinct pathways; either to methane and carbon dioxide by the aceticlastic methanogens (*Methanosaeta* and *Methanosarcina*), or to hydrogen and carbon dioxide by syntrophic acetate oxidizing bacteria (SAOB). Hydrogenotrophic methanogens further utilize the hydrogen and carbon dioxide to produce methane^4^. The aceticlastic methanogens are generally considered more sensitive for high ammonia levels than the SAOB and hydrogenotrophic methanogens. At high ammonia concentrations, a switch is therefore expected from aceticlastic methanogenesis to syntrophic acetate oxidation (SAO) and hydrogenotrophic methanogenesis. Recent research indicates that the tolerance to ammonia varies among hydrogenotrophic methanogens and SAOB strains^8^. Also, *Methanosarcina* has been observed to be rather robust to high ammonia levels in some studies^9–11^. The responses in complex methanogenic consortia to high ammonia concentrations are still poorly understood. Only a few SAOBs have been isolated and characterized so far, including two mesophilic bacteria (*Clostridium ultunense* ^12^ and *Syntrophaceticus schinkii* ^13^), the thermotolerant *Tepidanaerobacter acetatoxydans* ^14^ and three thermophilic SAOBs (strain AOR^15^, *Thermacetogenium phaeum* ^16,17^ and *Thermotoga lettingae* ^18^). The significance of these strains in methane producing anaerobic communities is not well described, and characterization of microbial communities by metagenomic approaches has suggested that also other SAOBs may exist in anaerobic biogas reactors^19,20^.

In the present study, we examined how temperature and ammonia concentrations affected the performance of high rate upflow anaerobic sludge bed (UASB) reactors treating pig manure slurry supernatant. We operated four reactors at two ammonia concentrations and at variable temperatures over a period of 358 days, and investigated effects on the methane production. For two of the reactors, the free ammonia concentrations reached as high as 1 g L^−1^. We characterized the bacterial and archaeal communities using Illumina sequencing of 16S rDNA amplicons, and aimed at identifying key players in methanogenesis at high ammonia concentrations.

## Results

### Reactor performance

**Figure 1:**
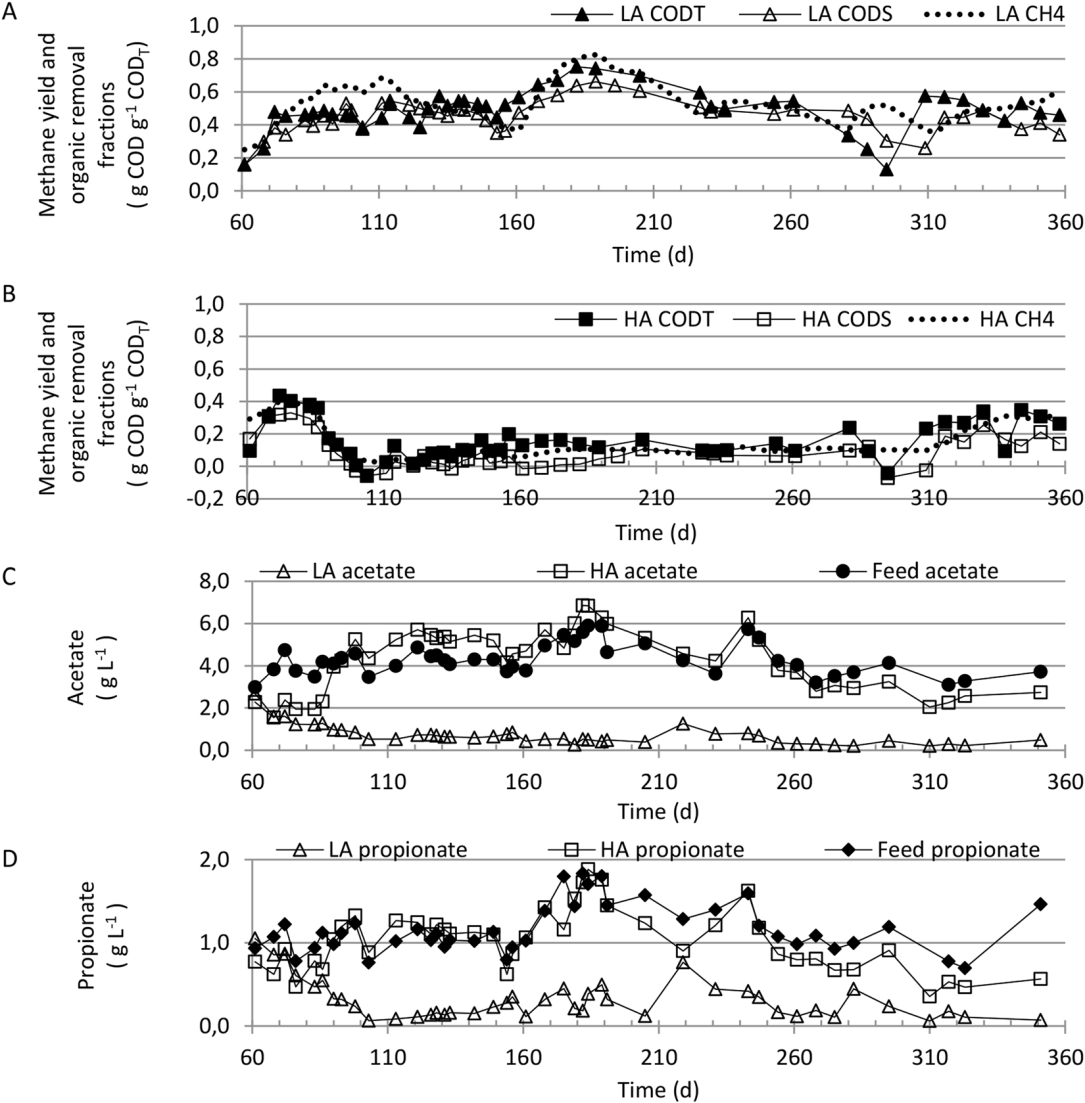
Performance of high ammonia (HA) and low ammonia (LA) rectors over the 358 days experimental period. Methane yield, total and soluble COD removal for (**A**) LA and (**B**) HA reactors, (**C**) acetate concentrations in the feed and effluents, and (**D**) propionate concentrations in the feed and effluents.

A total of four laboratory scale UASB reactors were studied for 358 days. The granules used as inoculum originated from a UASB reactor treating pulp and paper process wastewater. Whereas two of the reactors (LA1 and LA2; low ammonia concentration reactors) were fed untreated pig manure slurry supernatant throughout the experiment, the other two (HA1 and HA2; high ammonia concentration reactors) received manure slurry supernatant that had been added urea to increase the ammonia concentration in the reactors from day 69. The reactors produced biogas from the first day. Addition of urea to the feed for the HA reactors from day 69 resulted in a 2-fold increase in TAN and 20-fold increase in the FAN concentrations into the HA reactors compared to the LA reactors (Table 1). This, caused an approximately 2-fold increase in TAN and 6-fold increase in the FAN concentrations in the HA reactors compared to the LA reactors (Table S2). The average TAN concentrations were 3.7 ± 0.2 and 1.9 ± 0.1 g NH_4_^+^-N L^−1^ for the HA and LA reactors, respectively, whereas average FAN concentrations were 0.8 ± 0.2 and 0.14 ± 0.10 g NH_3_-N L^−1^ for the HA and LA reactors, respectively (Table S2). This resulted in a 10-fold drop of the methane yield from the HA reactors around day 100 (Fig. 1B). The yield in the LA reactors increased from 2.0 NL methane L^−1^ substrate to 3.7 during the same period.

**Table 1:** Properties of the pig manure slurry supernatant used as substrate. COD, acetate and propionate concentrations were the same for both influents. FAN was calculated from day 114 using equations given in Supplementary Information (Eq. S1-2). Total solids and volatile solids have been measured previously^21^.

Property	Average ± SD	
	LA influent	HA influent
TAN (g NH_4_^+^ – N L^−1^)	1.9 ± 0.2	3.7 ± 0.2
FAN (g NH_3_-N L^−1^)	0.06 ± 0.02	1.2 ± 0.3
pH	7.6 ± 0.2	8.7 ± 0.1
COD_T_ (g L^−1^)	16.6 ± 2.6	
COD_S_ (g L^−1^)	11.7 ± 1.3	
COD_VFA_ (g L^−1^)	8.8 ± 1.6	
Acetate (g L^−1^)	4.3 ± 0.8	
Propionate (g L^−1^)	1.2 ± 0.3

The COD_T_ removal became almost negligible (6%) and acetate accumulated in the HA reactors after urea addition (Fig. 1). Simultaneously, the COD_T_ removal increased to 49% in the LA reactors (Fig. 1A), and acetate concentration in the effluent decreased (Fig. 1C). The methane yield in the HA reactors increased slightly during the first 77 days of constant urea addition and stabilized at 0.53 ± 0.1 NL methane L^−1^ substrate during the next 150 days (*i.e*. days 146–296; Fig. 1B). This was accompanied by a slight increase in COD_T_ removal from approximately 14 to 18%, and a modest decrease in acetate concentration (Fig. 1). In the LA reactors, the biogas yield was seven times higher during the same 150 days period (3.7 ± 0.8 NL methane L^−1^ substrate), but with fluctuations due to different feed batches collected at the farm. Data from days 161–215 are unreliable because biogas was produced in the feed line due to high ambient temperatures and insufficient feed cooling. COD_T_ removal was 54 ± 6%, and the acetate conversion was almost complete, with a drop in concentration from 0.5 g L^−1^ on day 146 to 0.2 on day 296.

The imposed temperature reductions from 35 to 25 °C during the period from day 119 to 146 were accompanied by a 50% decrease in biogas production in the LA reactors, indicating a positive effect of operation at 35 °C. The methane yield did not, however, increase again towards the end of the experiment when the temperature was increased back to 35 °C, implying that the observed yield variations in the LA case are independent of temperature. This is supported by the quite constant and low volatile fatty acids (VFA) levels (Fig. 1C and D) after the start-up phase, independent of temperature levels. The observed yield variations and variations in COD_T_ removal are therefore attributed to variations in feed composition, to be expected since the feed was fresh pig manure from a barn with mainly farrows and wieners rearing^21^.

Towards the end of the experiment, the methane production, COD_T_, propionate, and acetate removal increased substantially in the HA reactors, and methane yields of around 1.8 NL methane L^−1^ substrate, and a COD_T_ removal of almost 60% was obtained (Fig. 1). This coincided with the temperature increase from 30 °C to 35 °C during days 296–322 (Fig. 1). However, this increase in methane yield cannot be explained by a temperature induced increase in growth rate alone^22^, indicating an adaption of the methanogenic consortium to the high ammonia concentration.

### Richness and diversity of microbial communities

Bacterial and archaeal communities were examined using distinct 16S rDNA primer pairs, and the resulting sequencing data were analyzed separately. A total of 672 907 and 1 318 803 reads were obtained with the bacterial and archaeal primers, respectively, after quality filtering and chimera removal (Table S1). OTU clustering and taxonomy assignment revealed that 22 of 2049 OTUs in the bacterial dataset belonged to Archaea and 820 of 923 OTUs in the archaeal dataset belonged to Bacteria. These were removed, and the two OTU tables subsequently yielded 2027 bacterial and 103 archaeal OTUs. The primers were designed to target highly conserved regions of the 16S rRNA bacterial and archaeal gene to maximize coverage and this probably resulted in reduced domain specificity. Comparisons of estimated richness (Chao1) and observed OTUs demonstrated that the sequencing effort across samples on average covered 70% and 92% of the estimated bacterial and archaeal richness, respectively. Thus, despite the high abundance of bacterial sequences in the archaeal data set, the diversity of the archaeal communities was well reflected at the obtained sequencing depth.

**Figure 2:**
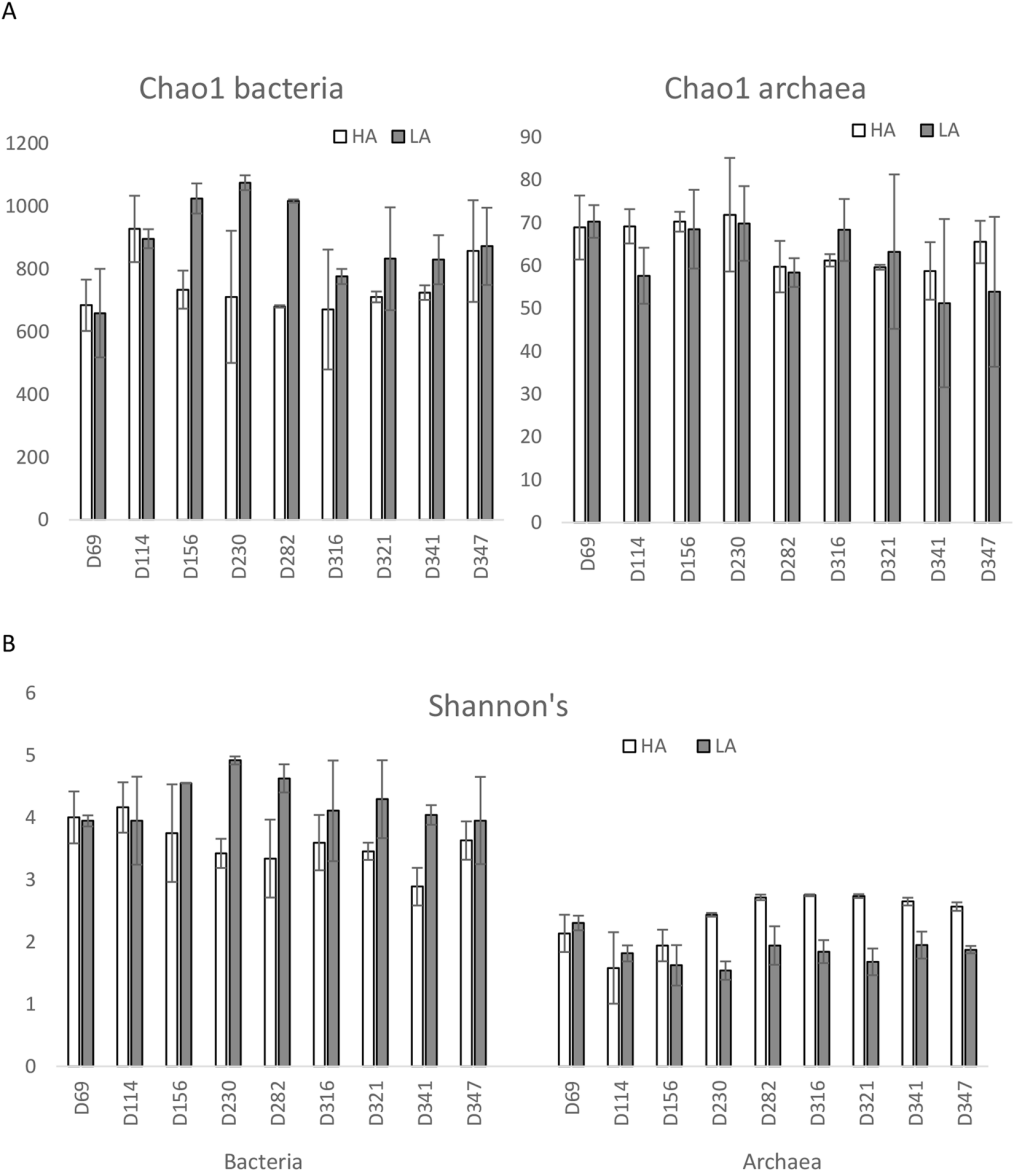
Diversity indices of bacterial and archaeal reactor communities. (**A**) Estimated richness (Chao1) and (**B**) Shannon’s diversity index given as average for two replicate reactors. HA and LA refers to the HA and LA reactors, and D refers to experimental day. Error bars represent standard deviation.

The Chao1 richness was around tenfold higher for bacterial than archaeal communities (Fig. 2A). Also, the Shannon’s diversity index was considerably higher for bacterial than archaeal communities (Fig. 2B). Increased ammonia concentration appeared to have opposite effect on the diversity of bacterial and archaeal communities: For bacteria, the Shannon’s diversity index was generally highest for the LA reactors, while for archaea, the HA reactor communities were more diverse (Fig. 2B).

### Overview of microbial community composition

**Figure 3:**
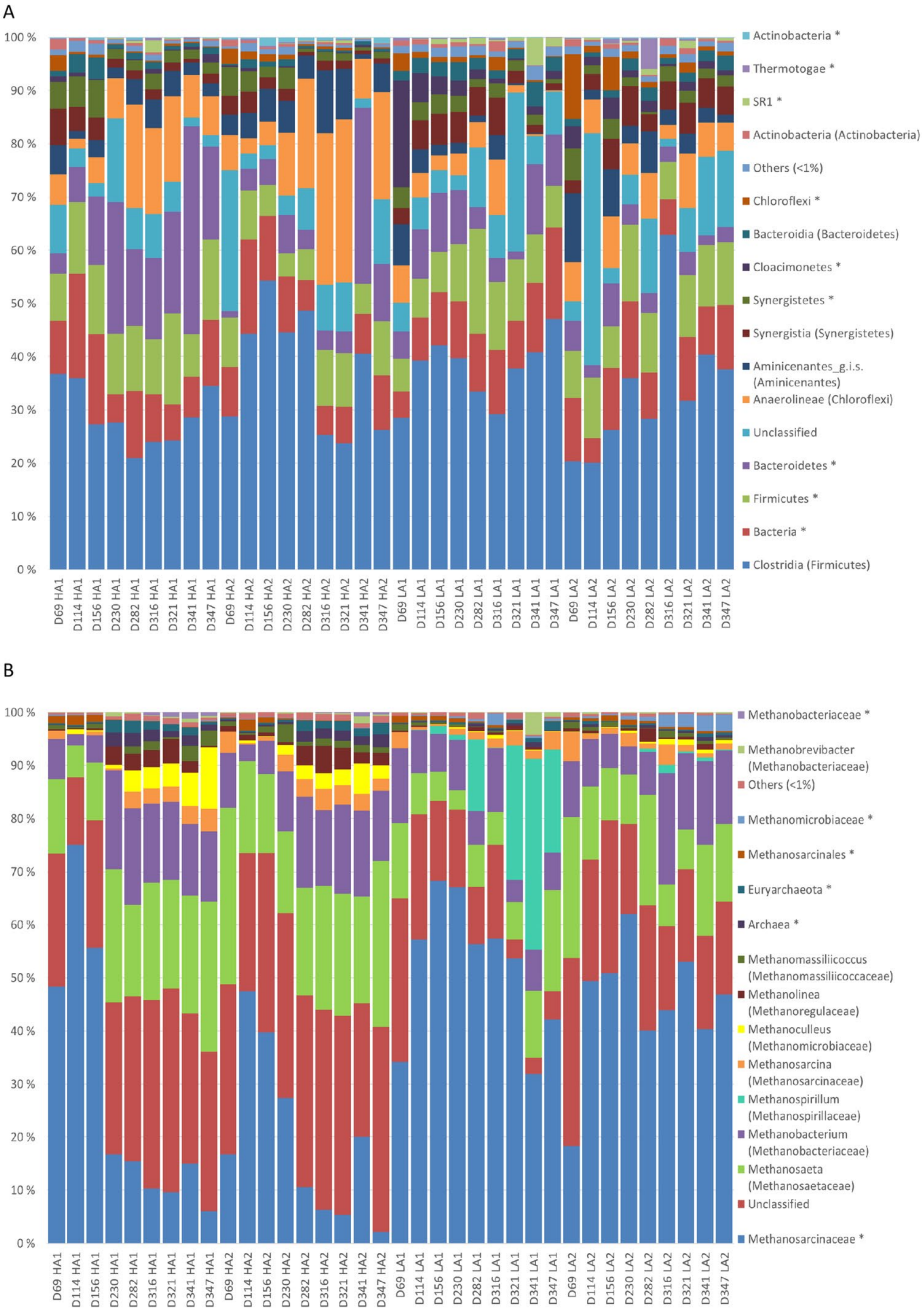
Relative abundances of bacterial classes (**A**) and archaeal genera (**B**) represented in the v3–4 16S rDNA amplicons obtained for individual reactor samples. Each bar represents one sample, and is labelled as follows: D indicates the experimental day; HA1 and HA2 refer to the two reactors operated at 3.7 ± 0.2 g NH_4_-N L^−1^, and LA1, and LA2 refers to the reactors operated at 1.9 ± 0.2 g NH_4_-N L^−1^. OTUs that could not be classified at the domain level are labeled “Unclassified”, while OTUs that could not be classified at class or genus level for bacteria and archaea, respectively, are labeled *. Only taxa represented by a portion of ≥1% of the sequence reads in at least one of the samples are shown. “Others” include all reads representing the taxa with <1% abundance in all samples.

Firmicutes was the most abundant bacterial phylum in all samples, and accounted for almost half of the bacterial sequence reads (44.8 ± 9.1%). A major fraction of these reads was classified as Clostridia at class level (Fig. 3A). In addition, the phyla Bacteroidetes, Cloroflexi, Synergistetes, Aminicenantes, Cloacimonetes, Actinobacteria, and Proteobacteria were observed in all reactor samples (Fig. 3A). A large number of OTUs was only classified as Bacteria or not even at domain level. Some of them were abundant, and they accounted for as much as 19.1 ± 8.2% of the reads on average. The most abundant of these OTUs (OTU_7) was suggested to represent Hydrogenedentes by UTAX and RDP Classifier, although with very low confidence scores (0.12 and 0.21, respectively). This OTU was particularly abundant in LA reactor samples, and accounted for as much as 37% of the reads in reactor LA2 at day 114. Other abundant unclassified OTUs were suggested to represent Clostridiales and Chloroflexi by both UTAX and RDP Classifier, but again with very low confidence scores.

**Figure 4:**
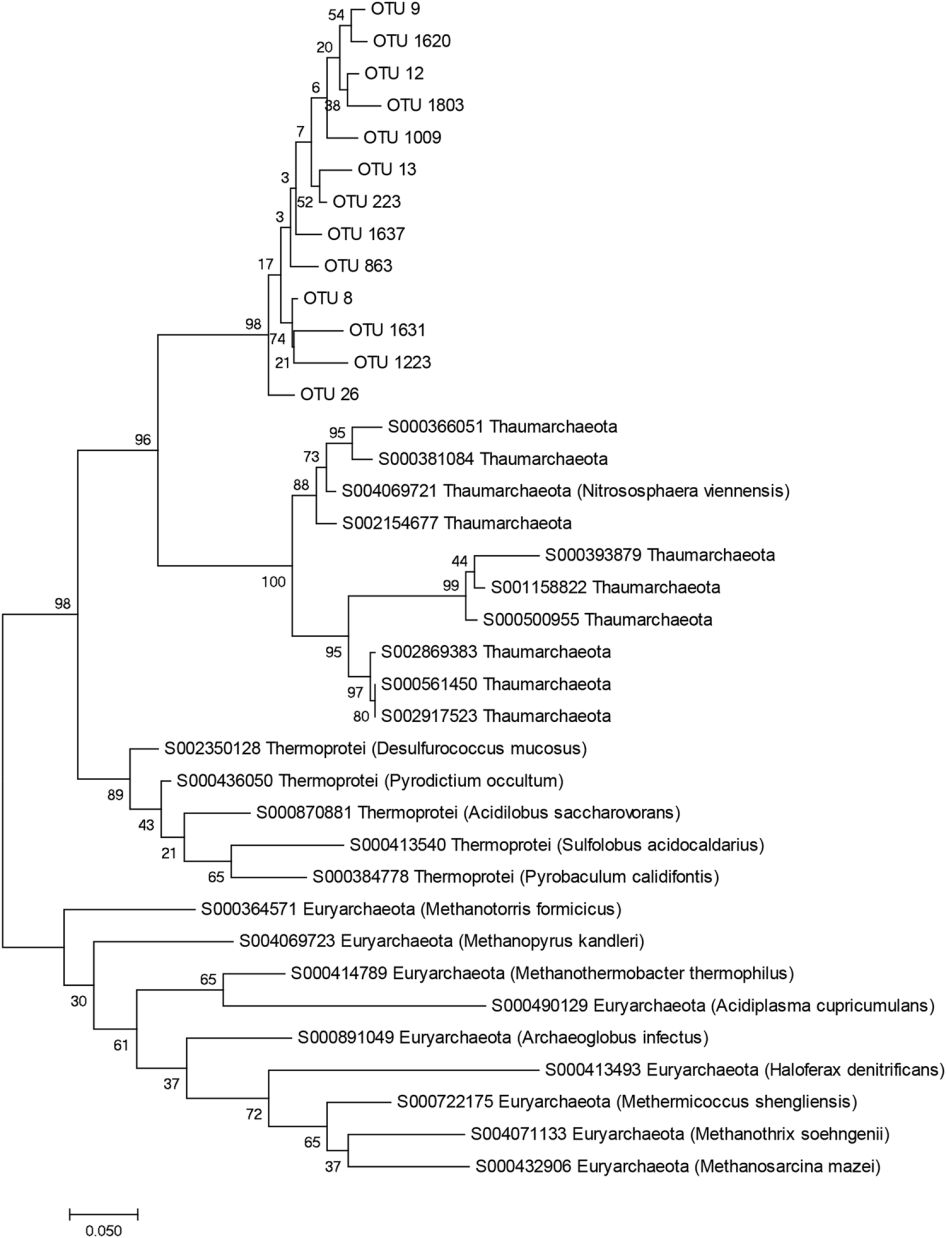
Maximum likelihood analysis including 13 OTUs identified in this study as potential relatives of Thaumarchaeota or Thermoprotei, and sequences representing Thermoprotei, Thaumarchaeota, and Euryarchaeota downloaded from the RDP database. For Thermoprotei and Euryarchaeota, “type strains” were selected. “Type strains” were not available for the Thaumarchaeota, but sequences representing both Nitrosopumilales and Nitrososphaerales were included. Number above nodes denote bootstrap percentages of 1000 replicates.

The archaeal communities were generally dominated by Euryarchaeotal taxa such as *Methanosarcinaceae*, *Methanosaeta*, and *Methanobacterium*, but the abundance clearly varied according to the ammonium concentration in the reactors (Fig. 3B). Crenarchaeota was observed in all samples, but never exceeded 1% of the reads in any of the samples. OTUs that could not be taxonomically assigned were highly abundant, particularly in the HA reactor samples (on average approximately 31 and 18% of the reads in HA and LA samples, respectively). Most of these reads were accounted for by 13 OTUs, which were suggested to represent Thaumarchaeota by the Utax classifier and Thermoprotei (Crenarchaeota) by the RDP Classifier, but with very low confidence scores. In sum, these 13 OTUs were abundant, particularly in the HA reactor samples, where they accounted for on average 28 ± 6% of the reads (from day 114, Fig. S1). We investigated these OTUs further by maximum likelihood analysis including 16S rDNA sequences classified as Thaumarchaeota, Thermoprotei, and Euryarchaeota (downloaded from the Ribosomal Database project, Fig. 4). The resulting phylogenetic tree indicated that these OTUs constituted a monophyletic clade, closer related to Thaumarchaeota than Thermoprotei (Fig. 4).

### Influence of operational conditions on community structure

**Figure 5:**
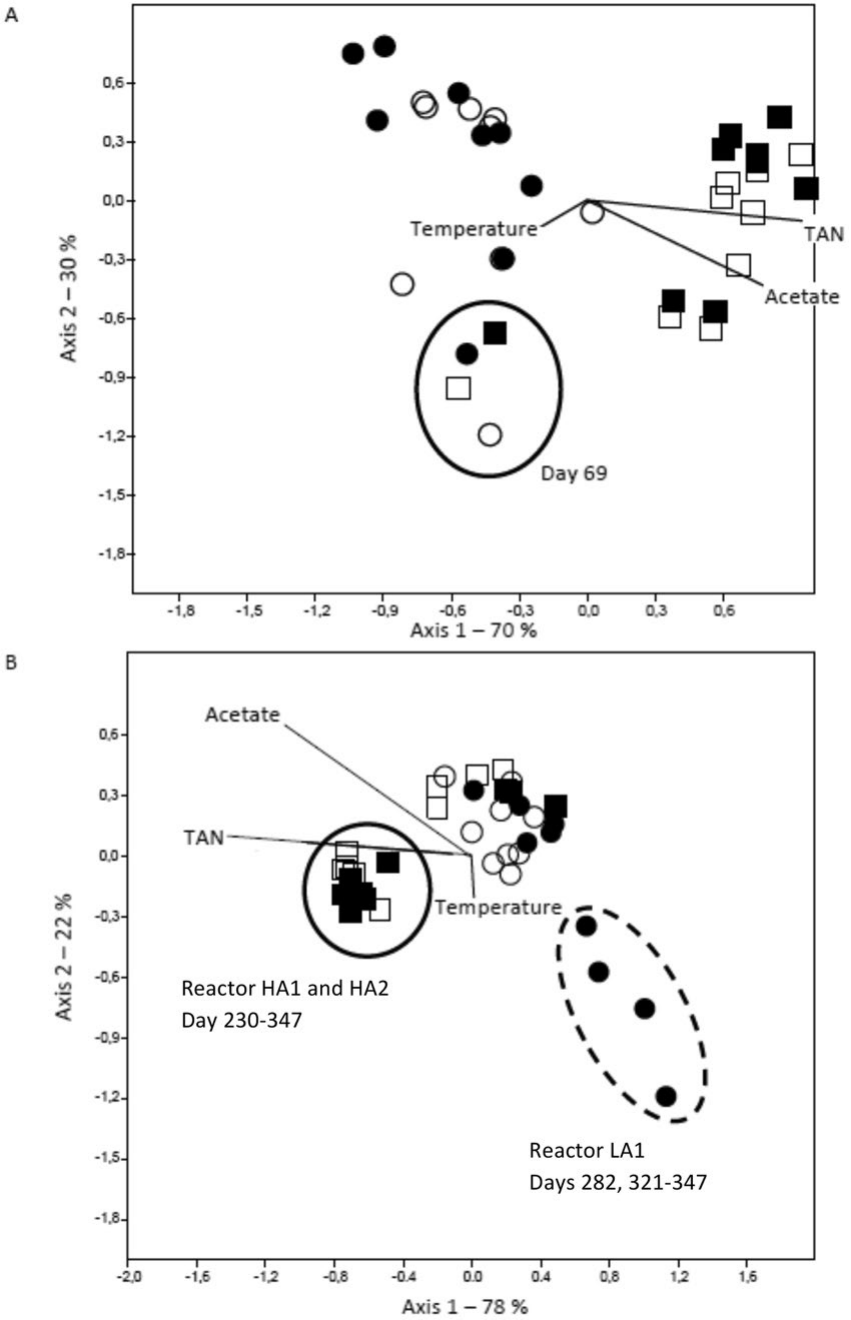
Canonical correspondence analysis with TAN, acetate and temperature as environmental variables, displayed as vectors in the plot, for bacterial (**A**) and archaeal (**B**) communities, for samples from reactor HA1 (■), HA2 (□), LA1(●) and LA2 (○).

A canonical correspondence analyses (CCA) was performed to examine the influence of environmental variables (temperature, TAN, and acetate concentration) on microbial community structure. TAN concentration obviously structured both bacterial and archaeal communities (Fig. 5). The CCA plot further suggested that TAN and acetate concentrations seems to play relatively equal roles in structuring the bacterial and archaeal communities, while temperature had less impact (Fig. 5).

Indeed, both bacterial and archaeal communities differed significantly between LA and HA reactors after day 114 (PERMANOVA, p < 0.0001). Whereas bacterial communities in LA and HA reactors appeared to diverge from each other already at day 114 (Fig. 5A), the archaeal HA and LA communities seemed to diverge later (from day 230; Fig. 5B). This indicates a slower response to the increased TAN concentration for the archaeal communities. SIMPER analysis for archaeal communities from day 230 and onwards showed that high abundance of the *Methanosarcinaceae* OTU_1 in LA (49.6% of the reads on average), and high abundance of the *Methanosaeta* OTU_2 in HA reactors (19.8% of the reads on average), explained as much as 46% of the Bray-Curtis dissimilarity between LA and HA archaeal communities (Table 2). The representative sequence of OTU_1 (*Methanosarcinaceae*) was classified as *Methanosarcina* by the RDP Classifier with high confidence, and the representative sequence for OTU_2 (*Methanosaeta*) was 100% identical to the 16S rRNA gene sequence of the type strain *Methanosaeta soehngenii* (S004071134), an obligate aceticlastic archaea. This indicates that *Methanosaeta*, but not *Methanosarcina*, was able to adapt to very high ammonia concentrations. OTU_1 and OTU_2 were the by far most abundant *Methanosarcina* and *Methanosaeta* OTUs, respectively, in the data set. The second most abundant *Methanosaeta* and *Methanosarcina* OTUs did not appear to be affected by the increased TAN concentration, but their abundances were much lower than those for OTU_1 and OTU_2 (Figs S2 and S3). Furthermore, three OTUs included in the non-methanogenic OTU cluster (see above), and two OTUs representing the hydrogenotrophic genera *Methanobacterium* and *Methanoculleus* were more abundant in the HA reactors (SIMPER, Table 2).

**Table 2:** The OTUs contributing most to the Bray-Curtis dissimilarity between LA and HA reactor communities from Day 230 to 347 as identified by SIMPER analysis.

OTU ID (taxonomy)	Cumulative	Mean abundance (%)	
Archaea	contribution (%)	HA	LA
OTU_1 (*Methanosarcinaceae*)	36.8	12.1	49.6
OTU_2 (*Methanosaeta*)	46.0	18.0	8.9
OTU_14 (*Methanospirillum*)	54.0	0.0018	8.2
OTU_3 (*Methanobacterium*)	59.8	14.7	10.2
OTU_12 (Unclassified)*	64.3	5.9	1.4
OTU_9 (Unclassified)*	68.7	6.0	1.6
OTU_13 (Unclassified)*	72.8	5.3	1.2
OTU_10 (*Methanoculleus*)	76.4	4.1	0.5
**Bacteria**			
OTU_3 (Bacteroidetes)	10.41	14.9	0.1
OTU_1 (*Clostridiaceae_1*)	18.55	12.4	4.9
OTU_2 (Anaerolineae)	26.40	15.4	4.5
OTU_9 (*Syntrophomonadaceae*)	33.15	2.5	11.4
OTU_7 (Unclassified/Hydrogenedentes)**	37.27	0.0016	5.9
OTU_12 (Unclassified/Chloroflexi)**	39.78	4.17	0.8
OTU_6 (*Syntrophomonadaceae*)	42.19	4.9	2.5
OTU_19 (Clostridiales)	43.92	2.7	0.2

*The unclassified archaeal OTUs were part of the non-euryarchaeotal archaea cluster (see Fig. 4). **The unclassified bacterial OTUs were suggested to be Hydrogenedentes (OTU_7) and Chloroflexi (OTU_12), but with low confidence thesholds.

The methane yield in the HA reactors were low until it started increasing from around day 300 (Fig. 1A), and the OTUs identified in SIMPER analysis as important differences between the HA and LA archaeal communities were not necessarily responsible for the increased methane production towards the end of the experiment. Spearman correlation analysis, including only HA reactor samples from day 230 to 347, identified a strong negative correlation between acetate concentration and biogas yield in the HA reactors (p = 2∙10^−9^). Interestingly, the *Methanosaeta* OTU_2 identified in the SIMPER analysis (Table 2) was one of four archaeal OTUs that were positively correlated to methane yield (p = 0.01). Furthermore, three OTUs representing hydrogenotrophic methanogens (*Methanoculleus* OTU_10, *Methanobacteriaceae* OTU_57, and *Methanobrevibacter* OTU_1142, see Table S3) were positively correlated with methane yield in the HA reactors (p < 0.05). Abundances of these OTUs are given for all reactor samples in Table S3.

The archaeal communities developed differently in the LA1 and LA2 reactors, even though they were operated under the same conditions and had similar methane yields. The most striking difference was the presence of an OTU representing *Methanospirillum* in LA1 in abundances up to 36% (Fig. 2B). In LA2, *Methanospirillum* never exceeded 1.6%, while in the HA reactors, it was barely detected (<0.006%). The increase of *Methanospirillum* in LA1 coincided with a lower abundance of unclassified OTUs compared to the other reactors (Fig. 3B).

For the bacterial communities, an OTUs representing *Syntrophomonadaceae* (OTU_9) was the most abundant one in the LA reactor samples, accounting for on average around 9% of the reads from day 230 and onwards. The second most abundant bacterial OTU (OTU_7; Table 2) in the LA reactors was one of the unclassified OTUs that might represent Hydrogenedents (see above). This OTU was very rare in the HA reactor communities (Table 2). In the HA reactors, three  bacterial OTUs, representing *Clostridiaceae_1*, Anaerolinea, and Bacteroidetes (OTU_1, OTU_2, and OTU_3, respectively; see Table 2), accounted for as much as 40% of the bacterial reads on average (from day 230). The RDP Classifier tool classified OTU_3 as *Clostridium sensu stricto* (confidence threshold 0.95). The most abundant bacterial OTUs (OTUs 1, 2, 3, 7, and 9) also contributed most to the Bray-Curtis dissimilarity between the HA and LA reactor bacterial communities (SIMPER, Table 2).

To identify bacterial OTUs associated with the increased methanogenic activity in the HA reactors towards the end of the experiment, we performed a Spearman correlation analysis, including the HA reactor samples from day 230 to 347. Three bacterial OTUs, representing *Syntrophomonadaceae* (OTU 9; Table 2), Synergistia, and Cloacimonetes, were positively correlated to methane yield. The abundance of the *Syntrophomonadaceae* OTU increased in HA reactors towards the end of the experiment, but never reached the same abundance in the HA as in the LA reactor samples (on average 2.5 and 11.4% of the reads for the HA and LA samples, respectively; Table 2). Of these three OTUs, only the Cloacimonetes OTU was more abundant in HA than LA reactors, though it never accounted for more than 0.59% of the reads in any of the HA communities. Abundances for these bacterial OTUs are given for all reactors samples in Table S3.

We examined the bacterial OTU table for the presence of genera including previously described syntrophic acetate oxidizing bacteria (SAOBs). The bacterial OTU table contained four OTUs classified as *Syntrophaceticus* or *Tepidanaerobacter*. These genera include the SAOBs *S. schinkii* and *T. acetatoxydans*. One of the *Tepidanaerobacter* OTUs was highly similar (98% of 402 nucleotide positions) to the *T. acetatoxydans* 16S rDNA sequence (GenBank accession number HF563609). The highest abundance for this OTU was found in the HA1 reactor at day 347, where it accounted for 0.16% of the reads, but it was rare in all other samples ( < 0.08%). One of the *Syntrophaceticus* OTUs was identical to the *S. schinkii* type strain 16S rDNA sequence (accession number EU386162). However, it was rarely observed in the HA reactor communities (maximum 0.01% of the reads), and was more abundant in reactor LA2, but never exceeded 0.09% of the reads in any of the samples. Furthermore, we searched our data set for OTUs with high similarity to the 16S rRNA sequence of the SAOB identified by Frank *et al*.^19^, but observed maximum 79% sequence identity. Hence, we found no indications that previously described SAOBs were associated with the methane production in the HA reactors.

## Discussion

Urea was added to the feed to the HA reactors from experimental day 69 to increase the TAN concentrations. Due to the lack of pH control, the pH increased to around 8.5, resulting in very high concentrations of free ammonia (around 0.83 g L^−1^ in HA reactors, Table S2). After a dramatic decrease in the methane yield on the HA reactors, the methane production stabilized at around 0.53 ± 0.1 NL methane L^−1^ in the period from experimental day 146 to 296 (Fig. 1). This increased methane production coincided with the 10 °C reduction in temperature (experimental day 119–146). This may be due to ammonia inhibition being less severe at lower temperatures, as previously described by Rajagopal, *et al*.^23^ and quantified as an inhibition factor in the anaerobic digestion model1 (ADM1) by Batstone, *et al*.^24^. The NH_3_ inhibition factor, using TAN = 3554 g L^−1^ and pH = 8.55 for this case, is 0.03 at 35 °C and 0.05 at 25 °C, where zero is complete inhibition and 1 implies no inhibition, so in this case it should be almost completely inhibited. The difference is a result of the acid dissociation constant K_a_ changing with temperature. The temperature effect on inhibition is evidently slightly larger than the effect on growth rate since biogas production increased when the temperature decreased (Fig. 1A). Both acetate and propionate removal was poor, and tended to accumulate in the HA reactors in the period from around day 100 to 236, indicating inhibition of acetate and propionate oxidation. Both acetate and propionate accumulations are indicators of process instability^25,26^.

The strong increase in biogas production (approximately 200%) observed in the HA reactors towards the end of the experiment coincided with a temperature increase from 25 to 35 °C. However, this large increase in methane yield cannot be explained by a temperature induced growth rate increase alone, according to estimates done as described by Henze and Harremoes^22^. Acetate and propionate removal improved considerable from around day 300, at FAN concentrations as high as 1 g L^−1^. Thus, the ammonia inhibition appeared to be much reduced at the end after around 200 days of adaptation, allowing methane production at a rather extreme FAN level.

The LA reactors produced methane at relatively high yields throughout the experiment. Effects of mesophilic temperature variations between 25 and 35 °C on the methane production was examined. Previous studies on this question have shown discordant results; some report a positive correlation between temperature and methane yield, some find no temperature effects, while yet others demonstrated negative correlations, probably due to increased levels of free ammonia^27^. Here, we found a reduction in methane yield accompanying the temperature reduction from 35 and 25 °C in the period from day 119 to 146. However, the temperature increase from day 296 until the end of the experiment did not result in increased methane yield. During this period, because nearly all acetate and propionate was converted, the methane yield was probably close to maximum, and could not be improved further.

Diversity is generally found to be higher for Bacteria than Archaea in anaerobic digestion^28^. Here we found that the OTU richness was as much as 10-fold higher for bacterial compared to archaeal communities. Still, we found relatively high archaeal diversity, with the presence of around 10 relatively abundant methanogenic genera (Fig. 3B). Hagen, *et al*.^29^ found that the archaeal communities in biogas reactors operated at relatively high FAN concentrations were almost completely dominated by *Methanothermobacter*, with a minor fraction of *Methanosaeta* (around 1% of the reads). Also Ziganshina, *et al*.^30^ found that the archaeal communities in biogas reactors with high TAN concentrations were dominated by a few genera; *Methanosarcina* accounted for around 80–90% of the archaeal reads, while *Methanoculleus* accounted for 10–16%. We aimed at designing broad coverage archaeal 16S rRNA PCR primers, and apparently succeeded in obtaining a detailed characterization of the archaeal communities. It was also interesting to note that the archaeal communities appeared to become more diverse in response to increased TAN concentrations (measured as Shannon’s diversity index, Fig. 2B). This is surprising, because environmental disturbances previously has been found to reduce microbial community diversity and functional stability^31^.

We identified a cluster of unclassified, non-euryarchaeotal OTUs that were abundant in all samples, but particularly in the HA reactors. Here, they accounted for as much as around 30% of the archaeal reads for most samples. In phylogenetic analysis, these OTUs clustered together in a distinctive group, related to Nitrosopumilales and Nitrososphaerales (Thaumarchaeota, Fig. 4), which include ammonia oxidizing archaea (AOA). These non-euryarchaeal OTUs were obviously ammonia tolerant. However, as part of an anaerobic consortium, they were not likely to oxidize ammonia. The AOAs are generally aerobic, although some appear to thrive at low oxygen concentrations^32,33^. Archaeal taxa that do not represent described methanogens classified as Euryarchaeota have previously been observed in biogas reactors, and has often been referred to as Crenarchaeota^34,35^. Chen and He^36^ recently performed a phylogenetic analysis of previously published sequences that had been assumed to represent non-methanogenic archaea in anaerobic reactors. They found that they constituted a single phylogenetic clade related to, but distinct from, Thaumarchaeota, similar to the OTU cluster identified in our study. Chen and He^36^ referred to these as non-methanogenic archaea (NMA), and a more detailed analysis suggested that they were placed within the Group I.3 archaeal lineage, together with uncharacterized archaeal populations found in environments like flooded soils and sediments^36^. Chen and He^36^ performed batch experiments and demonstrated that acetate promoted growth of NMA (compared to formate, propionate, butyrate, and methanol), but still their relative abundance of NMA was low (around 1%) compared to the aceticlastic methanogens (relative abundances of around 80%). Recently, methanogenic archaea not classified as Euryarchaeota have been identified. Vanwonterghem, *et al*.^37^ described the existence of divergent methylcoenzyme M reductase genes recovered from anoxic environments, and suggested these genomes represented a new phylum, *Candidatus* Verstraetearchaeota. Moreover, Evans, *et al*.^38^ recovered two genomes belonging to the Bathyarchaeota from a deep aquifer containing functional genes needed for methylotrophic methanogenesis. More studies are needed to map the phylogenetic distribution of methanogens among Archaea. We cannot exclude the possibility that the abundant non-euryarchaeal OTU cluster identified in our samples may represent methanogens.

Although we found the highest abundances of the non-euryarchaeotal OTU cluster in the HA reactors, it was generally abundant, accounting for around 15–30% of the reads for most samples (Fig. S1). The noteworthy exceptions were the LA1 samples, which were characterized by a high abundance of *Methanospirillum* which coincided with a decrease in abundance of the non-euryarchaeotal OTU cluster. Two described *Methanospirillum* species, *M. hungatei* ^39^ and *M. stamsii* ^40^, are strict anaerobic, hydrogenotrophic methanogens, utilizing hydrogen or formate and CO_2_ for methanogenesis. *M. stamsii* require acetate for carbon supply. One possible interpretation of these observations is that *Methanospirillum* and the non-euryarchaeotal OTU cluster compete in the same niche. The abundances of *Methanospirillum* and the non-euryarchaeotal OTU cluster abundances differed considerably between the LA reactors, although they had been operated at the same conditions and had similar methane yields. This may indicate the existence of functional redundancy and a possible influence of random processes in the methanogenic community assembly.

Previous studies indicate that stressful conditions, such as increased ammonia levels, favor hydrogenotrophic methanogens and a shift from aceticlastic methanogenesis to bacterial syntrophic acetate oxidation coupled with hydrogenotrophic methanogenesis^4,28^. However, Fotidis, *et al*.^41^ observed no shift to SAO after exposure of a mesophilic, acclimated methanogenic community to ammonium at high concentrations (TAN of 7 g L^−1^, but considerable lower FAN concentrations), but found that the aceticlastic *Methanosarcina* was the dominant methanogen. Generally, *Methanosarcina* has been found to be more robust to TAN concentrations up to 4–6 g L^−1^ than *Methanosaeta* ^9–11^. This has been explained by the fact that *Methanosarcina* cells form clusters, which may provide protection by reducing the diffusion of ammonia into the cells^42^.

Surprisingly, we found that a *Methanosaeta* OTU, with a 16S rRNA gene sequence identical to the type strain of the obligate aceticlastic *Methanosaeta soehngenii*, was positively correlated to methane yield in the HA reactors towards the end of the experiment. It was relatively abundant in the HA reactors throughout the experiment, but increased towards the end (Fig. S2). This is a strong indication that *Methanosaeta* can perform acetate conversion even at FAN levels around 1 g L^−1^. In contrast to the studies referred to above, we found that *Methanosaeta* was more abundant than *Methanosarcina* at high FAN concentrations, and *Methanosarcina* levels dropped markedly, from around 60% to below 10% of the reads from around day 282 (Fig. S3). Calli, *et al*.^9^ observed that the *Methanosarcina* cell clusters disintegrated at FAN levels above 0.6 g L^−1^. Thus, *Methanosarcina* might not be protected by clustered growth at FAN levels as high as in the HA reactors. Loss of *Methanosaeta* activity at FAN over 100 mg/L has been associated with the loss of filamentous growth^42^. A possible explanation for the apparent high ammonia tolerance we observed for *Methanosaeta* in this study could be protection obtained by growing in aggregates with other microbes in the sludge granules. Zheng, *et al*.^43^ found that *Methanosaeta concilii* was positioned in the core of sludge granules, enclosed by syntrophic consortia, and with filamentous bacteria in the surface layer.

We were not able to identify previously described SAOB in the bacterial communities in our data set, but lately new SAOBs have been identified^19,20^ and we cannot exclude the existence of SAOB in the methanogenic communities examined here.

In the LA reactors, the most abundant OTU represented *Syntrophomonadaceae*. It increased in all reactors (both LA and HA) throughout the experiment (Table S3) and was positively correlated with methane yield in the HA reactors. This indicates that *Syntrophomonadaceae* probably was important for efficient methane production. Members of the *Syntrophomonadaceae* perform β-oxidation of carboxylic acids of four carbons or more, typically butyrate, and produce acetate and H_2_ in a reaction which is thermodynamically favorable only when H_2_ is maintained at low concentrations by syntrophic partners like hydrogenotrophic methanogens^44^. The second most abundant OTU (OTU_7) in the LA reactors could only be classified at the domain level, but might be related to Hydrogenedentes (see Results). A recent study^45^ suggests Hydrogenedentes may be involved in syntrophic degradation of glycerol and lipids in anaerobic consortia, but whether this is relevant for the functional role of OTU_7 is not known. OTUs representing *Clostridiaceae_1* (probably *Clostridium sensu stricto*), Anaerolinae, and Bacteroidetes dominated the bacterial communities in the HA reactors, accounting for almost 40% of the bacterial reads on average after Day 230. *Clostridium* species are known to produce hydrogen through the fermentation of organic substrates^46^. Bacteroidetes are able to ferment polysaccharides, and has been suggested to be involved in hydrolysis of cellulose in anaerobic digestion^47^. Anaerolineae has been found to be abundant in anaerobic digestive communities previously^48,49^. Some Anaerolineae species degrade carbohydrates in cooperation with hydrogenotrophic methanogens, and have been described as “semi-syntrophic”^50^. It was interesting to note that a bacterial OTU representing Cloacimonetes, although at low abundances (OTU_95, Table S3), was positively correlated to methane yield in the HA reactors. The increase in methane coincided with increased propionate removal. Cloacimonetes includes strains able to syntrophically oxidize propionate, such as Candidatus *Cloacamonas acidaminovorans* ^51^. We inspected the OTU table for taxa previously known to include syntrophic propionate oxidizing bacteria, such as *Syntrophobacter* and *Desulfobulbus* (Betaproteobacteria), *Pelotomaculum* and *Desulfotomaculum* (*Peptococcaceae*, Clostridia), and Atribacteria^52–55^. However, OTUs representing these taxa were generally rare in all reactor samples. Thus, we were not able to suggest other likely candidates for the increased propionate removal in the HA reactors towards the end of the experiment.

In summary, ammonia proved to be a major selective factor of bacterial and archaeal community structure. TAN concentrations of 3.7 g L^−1^ (FAN around 0.8 g L^−1^) inhibited methanogenesis in the HA UASB reactors fed particle rich pig manure supernatant. After almost 200 days of adaptation, acetate and propionate removal and methane production improved substantially. Abundant archaeal and bacterial OTUs, accounting for a large fraction of the reads obtained by Illumina sequencing of 16S rRNA amplicons, could not be taxonomically assigned below domain level. This illustrates that abundant members of anaerobic digestive consortia remain to be described. Phylogenetic analysis suggested that a group of such archaeal OTUs, highly abundant at high TAN concentrations, constituted a monophyletic group related to Thaumarchaeota. Previously described syntrophic acetate oxidizing bacteria were only observed sporadically and at low abundances in the HA reactors. The most abundant bacterial OTU in LA reactors represented *Syntrophomonadaceae*. This OTU was positively correlated to methane yield in the HA reactors, indicating that it was important for efficient methane production under ammonia stress. *Methanosaeta* was abundant, and was positively correlated to methane yield in the HA reactors, whereas *Methanosarcina* was more abundant in the LA reactors operated at TAN concentrations around 1.9 g L^−1^. In conclusion, efficient methane production, probably involving aceticlastic methanogenesis mediated by *Methanosaeta*, took place in the UASB reactors at FAN concentrations as high as 1 g L^−1^.

## Material and Methods

### Reactor influent and inoculum

The manure substrate was collected from a pig production farm in Porsgrunn, Norway. Pig manure handling has been described previously^2^ while the substrate properties are given in Table 1. After collection, the manure substrate was stored at 4 °C until use. A total of four laboratory scale reactors were studied for 358 days. Whereas two of the reactors (LA1 and LA2; low ammonia concentration) were fed untreated pig manure slurry supernatant throughout the experiment, the other two (HA1 and HA2; high ammonia concentration) received manure slurry supernatant that had been added urea (4 g L^−1^) to increase the ammonia concentration in the reactors during experimental days 69–358. Increasing amounts of urea were added during days 69–107, and from day 107 4 g L^−1^ urea was added to the HA substrate for the rest of the experiment. This resulted in increased total ammonia nitrogen (TAN) concentrations in the substrate for HA reactors (3.7 ± 0.2 g NH_4_-N L^−1^) compared to that for the LA reactors (1.9 ± 0.2 g NH_4_-N L^−1^) (Table 1). The pH of the untreated supernatant was 7.6 ± 0.2 during the whole period, while the pH increased to 8.7 ± 0.1 in the feed added urea. Due to the lack of pH control, the addition of urea resulted in an approximately 20 fold increase in the concentration of free ammonia nitrogen (FAN) in the supernatant fed to the HA reactors, reaching as high as 1.2 ± 0.3 g NH_3_-N L^−1^ (Table 1).

The granules used as inoculum originated from a UASB reactor treating pulp and paper process wastewater at “Norske Skog Saugbrugs” in Halden, Norway^2^. The granular inoculum had been stored at 11 °C prior to the experiment. The reactors were filled with approximately 180 ml of granules at the start of the experiment. Suspended solids and granules were separated inside the reactors to retain biomass.

### Reactor design and operation

Lab-scale process lines were set up utilizing identical pulse fed 370 mL UASB reactors with a liquid volume of 360 mL. The reactor design and measurements of COD_T_ (total COD), COD_S_ (soluble COD), pH, VFA, NH_4_^+^-N, gas composition and methane production has been described previously^2^. The reactors were all operated at a hydraulic retention time (HRT) of 1.0 day. The organic loading rate (OLR) was 16 ± 2 g COD L^−1^ d^−1^ for the entire experiment. The reactors were fed intermittently, 25 mL each time with 14 feedings for each HRT. It is therefore reasonable to assume continuous flow in the mass balance analysis of the process.

The reactors were operated at 35 °C, 30 °C and 25 °C during the following periods: 35 °C from startup; 30 °C during days 119–146; 25 °C at days 146–296; 30 °C at days 296–322 and 35 °C again during days 322–358. Unintentionally the substrate was heated up in the “sand trap” separator before reaching the reactor at days 161–215 due to unusual high summer temperatures. Afterwards this substrate line separator was kept at 5–15 °C by cold water.

### Sampling, DNA extraction and PCR amplification

Samples were collected from each reactor on the following days of the experiment; 69, 114, 156, 230, 282, 316, 321, 341 and 347. The reactors were stirred before sampling from the effluent to ensure a homogenous sample. The samples were kept frozen during the experiment and thawed prior to DNA extraction. The liquid phase was removed as follows: Samples were centrifuged at 200 g for 10 minutes, the supernatant was discarded and the pellet resuspended in phosphate buffer saline (PBS, 1x). This was repeated twice before centrifugation at 4000 g for 10 minutes to remove liquid before DNA extraction.

Total DNA was extracted using the Power Soil DNA isolation kit (Mobio Laboratories Inc., Carlsbad, CA, USA) as described by the manufacturer. DNA concentration was measured by NanoDrop Spectrometer ND-1000 (NanoDrop Technologies, USA). PCR primers targeting 16S rDNA in both bacteria and archaea were designed to target conserved regions. Coverage was optimized by using alignments of sequences downloaded from the Ribosomal Database Project (RDP; http://rdp.cme.msu.edu) and the RDP tool Probematch^56^. The resulting primers amplified the v3–4 region of the 16S rRNA gene; B-338F (5′-CCTACGGGWGGCAGCAG) and B-805R (5′-GACTACNVGGGTATCTAAKCC) amplifying 467 base pairs (bp) for bacterial DNA, A-340F (CCCTAYGGGGYGCASCAG) and A-760R (GGACTACCSGGGTATCTAATCC) for archaeal DNA. Bacterial and archaeal amplicons were generated for all samples and the amplicons were indexed as explained by Goux, *et al*.^57^. PCR amplifications of the 16S rDNA was performed using Phusion Hot Start DNA polymerase (Thermo Scientific, Lithuania) with 0.6 μM of each primer, approximately 1 ng µl^−1^ DNA template, 0.8 mg ml^−1^ BSA and 2 mM MgCl_2_ for 30 cycles (98 °C 15 s, 50 °C 20 s, 72 °C 20 s). A second PCR for attachment of index sequences were performed as described by Goux, *et al*.^57^. PCR clean-up and normalization to obtain equimolar amplicon libraries (1 ng DNA µl^−1^) of each amplicon was performed using the SequalPrep Normalization Plate (96) Kit (Invitrogen, Maryland, USA) according to the manufacturer’s instructions. The bacterial and archaeal amplicons were pooled to generate one library and finally were concentrated to 15 ng µl^−1^ using Amicon Ultra Centrifugal Filter Units (Millipore, Ireland) as specified by the manufacturer. The samples analyzed in this study accounted for 36 of 116 uniquely indexed amplicons constituting the library that was sequenced on a MiSeq lane (Illumina, San Diego, CA) with v3 reagents employing 300 bp paired end reads at the Norwegian Sequencing Centre. PhiX library (Illumina) was blended to 50%. Data was processed using RTA 1.18.54 (Illumina). The resulting Illumina sequencing data were deposited at the European Nucleotide Archive (accession numbers ERS1982668-ERS1982721 and ERS1982739-ERS1982792).

### Data analysis and statistics

The Illumina sequencing data were processed with the high performance USEARCH utility (version 8.1.1825) (http://drive5.com/usearch/features.html). The processing was carried out as implemented in the UPARSE pipeline^58^. The major steps in the pipeline included demultiplexing, quality trimming, chimera removal, and clustering to obtain OTU tables at 97% similarity level. The subsequent taxonomy affiliation was based on the Utax script implemented in the UPARSE pipeline with a confidence value threshold of 0.8 and the RDP reference data set (version 15). In practical terms the pipeline was split at six break points where optimization was deemed likely and was supported by seven Perl scripts (available on request) to ease the processing. The Qiime pipeline^59^ was used for determining Chao1 richness and relative abundances at different taxonomic levels. The RDP tools Classifier^60^ and Sequence Match were used to analyze OTUs of particular interest.

Statistical analyses were performed using the program package PAST version 2.17^61^. Similarities between community profiles were calculated as Bray-Curtis similarities^62^. Differences in average Bray–Curtis similarities between groups of samples were tested using PERMANOVA^63^. SIMPER (Similarity Percentage) analysis was employed to identify OTUs responsible for differences (measured as Bray-Curtis similarities) between sample groups^64^. Canonical correspondence analysis (CCA) was employed to elucidate the relationship between microbial community composition in the samples and environmental variables^65^. To evaluate correlation between relative abundance of genera and environmental variables we used Spearman’s rank-order correlation coefficient to avoid the assumption of linear correlations. The probability of non-zero Spearmans’s correlation was computed using a t test. The analyses were performed at the OTU level, and all OTUs with minimum 0.5% abundance in at least one sample were included. A Maximum likelihood tree based on the Tamura-Nei model^66^ was inferred using MEGA 7^67^. Sequences representing Thermoprotei, Thaumarchaeota, and Euryarchaeota were retrieved from the Ribosomal Database Project^56^. Details are given in the legend of Fig. 4.

The reactors were operated at the University College of Southeast Norway, Porsgrunn, Norway, while the microbial analyses were executed at Norwegian University of Science and Technology, Trondheim, Norway.

## Electronic supplementary material

Supplementary information
