# Supplementary material for: Anaerobic digestion of pig manure supernatant at high ammonia concentrations characterized by high abundances of Methanosaeta and non-euryarchaeotal archaea
Source: Sci Rep. 2017 Nov 8;7:15077. doi: 10.1038/s41598-017-14527-1 (PMC5678120; doi:10.1038/s41598-017-14527-1)
Supplement: Supplementary file 1 — Supplementary information [file 41598_2017_14527_MOESM1_ESM.pdf]

**Supplementary information to the article titled**

# Anaerobic digestion of pig manure supernatant at high ammonia concentrations characterized by high abundances of *Methanosaeta* and non-euryarchaeotal archaea

Anna Synnøve Røstad Nordgård[a], Wenche Hennie Bergland[b], Olav Vadstein[a], Vladimir Mironov[a], Rune Bakke[b], Kjetill Østgaard[a] and Ingrid Bakke[a]

a. Department of Biotechnology and Food Science, Norwegian University of Science and Technology (NTNU), Sem Sælands vei 6/8, 7491 Trondheim, Norway
b. Department of Process, Energy and Environmental Technology, University College of Southeast Norway (USN), Kjølnes ring 56, 3918 Porsgrunn, Norway.

anna.s.r.nordgard@ntnu.no, tel. + 47 73 59 16 47, + 47 472 44 895

wenche.bergland@hit.no, tel. +47 35 57 52 17, +47 928 27 207

olav.vadstein@ntnu.no, tel. +47 918 97 034

vladimir.mironov@ntnu.no, tel. +47 73 55 03 49

rune.bakke@hit.no, tel. +47 35 57 52 41, +47 986 24 908

kjetill.ostgaard@ntnu.no, tel. +47 73 59 40 68

Ingrid.bakke@ntnu.no, tel. +47 73 59 78 59, +47 932 47 872, Corresponding author

The reactors were operated at University College of Southeast Norway, Porsgrunn, Norway, while the microbial analyses were executed at Norwegian University of Science and Technology, Trondheim, Norway.

**Table S1.** Number of reads for each sample

| Exp. Day | Bacteria | | | | Archaea | | | |
|---|---|---|---|---|---|---|---|---|
| | HA1 | HA2 | LA1 | LA2 | HA1 | HA2 | LA1 | LA2 |
| D69 | 10498 | 9544 | 6697 | 6502 | 29021 | 27159 | 19375 | 29302 |
| D114 | 13826 | 14097 | 19166 | 22362 | 33338 | 10454 | 33946 | 19725 |
| D156 | 16931 | 20696 | 18045 | 16553 | 16378 | 26183 | 34419 | 14172 |
| D230 | 10867 | 18075 | 13388 | 13514 | 35544 | 39937 | 59669 | 36248 |
| D282 | 23593 | 24431 | 17596 | 18544 | 49475 | 26240 | 45905 | 28285 |
| D316 | 27045 | 9909 | 8653 | 11454 | 28431 | 42880 | 30555 | 28233 |
| D321 | 27553 | 21213 | 13641 | 15852 | 45634 | 38311 | 40409 | 37590 |
| D341 | 17891 | 23001 | 26136 | 17361 | 35470 | 36607 | 37843 | 32789 |
| D347 | 40816 | 29858 | 24563 | 34537 | 39812 | 37466 | 23900 | 45895 |

**Table S2**. Temperature, total ammonia nitrogen (TAN) and free ammonia nitrogen (FAN) concentrations in the reactor samples.

| Day | Temp. [°C] | Influent HA TAN [mg/L] | Influent HA FAN [mg/L] | Influent LA TAN [mg/L] | Influent LA FAN [mg/L] | HA1 TAN [mg/L] | HA1 FAN [mg/L] | HA2 TAN [mg/L] | HA2 FAN [mg/L] | LA1 TAN [mg/L] | LA1 FAN [mg/L] | LA2 TAN [mg/L] | LA2 FAN [mg/L] |
|---|---|---|---|---|---|---|---|---|---|---|---|---|---|
| 69 | 35 | 2030 | 153 | 2030 | 153 | 1910 | 186 | 1913 | 190 | 1845 | 187 | 1865 | 160 |
| 114 | 35 | 3490 | 1260 | 1935 | 58 | 3620 | 1086 | 3565 | 1104 | 1990 | 189 | 1935 | 169 |
| 156 | 25 | 3305 | 746 | 2015 | 53 | 3580 | 631 | 3450 | 637 | 2005 | 78 | 1980 | 84 |
| 230 | 25 | 3860 | 927 | 1950 | 60 | 4070 | 703 | 3990 | 690 | 2010 | 62 | 1980 | 65 |
| 282 | 25 | 3290 | 1237 | 1740 | 43 | 3470 | 600 | 3440 | 606 | 1700 | 101 | 1610 | 176 |
| 316 | 30 | 3500 | 990 | 1790 | 76 | 3690 | 850 | 3570 | 1078 | 1760 | 143 | 1920 | 166 |
| 321 | 30 | 3620 | 1315 | 1750 | 54 | 3690 | 1027 | 3670 | 1021 | 1770 | 200 | 1800 | 225 |
| 341 | 35 | 3680 | 1214 | 1780 | 76 | 3580 | 721 | 3700 | 732 | 1780 | 162 | 1800 | 145 |
| 347 | 35 | 3600 | 1557 | 1750 | 29 | 3860 | 980 | 3610 | 826 | 1810 | 162 | 1870 | 189 |

**Table S3.** Abundance of bacterial and archaeal OTUs in all reactor communities, found to be positively correlated to methane yield in HA samples (Spearman, $p<0.05$).

| | Bacteria | | | Archaea | | | |
|---|---|---|---|---|---|---|---|
| | OTU_9 Syntrophomonadaceae | OTU_38 Synergistia | OTU_95 Cloacimonetes | OTU_2 Methanosaeta | OTU_10 Methanoculleus | OTU_57 Methanobacteriaceae | OTU_1142 Methanobrevibacter |
| **D69 HA1** | 1.34 | 0.10 | 0.00 | 11.53 | 0.01 | 0.00 | 0.00 |
| **D69 HA2** | 0.00 | 0.08 | 0.00 | 24.05 | 0.01 | 0.00 | 0.02 |
| **D69 LA1** | 0.78 | 0.07 | 0.00 | 11.54 | 0.00 | 0.00 | 0.02 |
| **D69 LA2** | 0.03 | 0.06 | 0.00 | 21.43 | 0.01 | 0.00 | 0.01 |
| **D114 HA1** | 0.71 | 1.01 | 0.00 | 5.37 | 0.83 | 0.00 | 0.00 |
| **D114 HA2** | 0.04 | 0.50 | 0.00 | 11.69 | 0.57 | 0.00 | 0.00 |
| **D114 LA1** | 2.68 | 0.38 | 0.00 | 5.91 | 0.05 | 0.00 | 0.03 |
| **D114 LA2** | 0.35 | 0.26 | 0.00 | 10.47 | 0.07 | 0.00 | 0.09 |
| **D156 HA1** | 0.02 | 0.71 | 0.04 | 9.80 | 0.28 | 0.00 | 0.00 |
| **D156 HA2** | 0.00 | 0.21 | 0.01 | 13.02 | 0.05 | 0.00 | 0.00 |
| **D156 LA1** | 2.48 | 1.15 | 0.00 | 4.24 | 0.12 | 0.00 | 0.00 |
| **D156 LA2** | 0.40 | 0.61 | 0.00 | 7.05 | 0.13 | 0.00 | 0.00 |
| **D230 HA1** | 0.64 | 0.22 | 0.01 | 16.45 | 0.71 | 0.00 | 0.13 |
| **D230 HA2** | 0.07 | 0.22 | 0.19 | 13.79 | 1.82 | 0.01 | 0.01 |
| **D230 LA1** | 1.37 | 0.78 | 0.00 | 2.74 | 0.26 | 0.00 | 0.02 |
| **D230 LA2** | 2.26 | 0.75 | 0.00 | 6.27 | 0.41 | 0.00 | 0.04 |
| **D282 HA1** | 1.14 | 0.45 | 0.06 | 13.62 | 4.03 | 0.03 | 0.05 |
| **D282 HA2** | 0.77 | 0.14 | 0.03 | 13.46 | 2.64 | 0.00 | 0.03 |
| **D282 LA1** | 6.85 | 0.73 | 0.00 | 6.08 | 0.21 | 0.00 | 0.05 |
| **D282 LA2** | 9.22 | 0.17 | 0.01 | 15.00 | 0.45 | 0.00 | 0.08 |
| **D316 HA1** | 4.71 | 0.46 | 0.59 | 17.76 | 4.09 | 0.52 | 0.03 |
| **D316 HA2** | 0.88 | 0.23 | 0.04 | 17.83 | 2.98 | 0.17 | 0.02 |
| **D316 LA1** | 5.14 | 0.25 | 0.02 | 4.94 | 0.67 | 0.00 | 0.03 |
| **D316 LA2** | 2.20 | 0.09 | 0.04 | 6.59 | 0.99 | 0.00 | 0.02 |
| **D321 HA1** | 7.59 | 0.24 | 0.14 | 16.47 | 4.35 | 0.85 | 0.10 |
| **D321 HA2** | 1.28 | 0.23 | 0.13 | 16.78 | 2.89 | 0.19 | 0.13 |
| **D321 LA1** | 13.46 | 0.41 | 0.00 | 5.23 | 0.22 | 0.00 | 0.04 |
| **D321 LA2** | 5.62 | 0.28 | 0.06 | 6.21 | 0.98 | 0.00 | 0.01 |
| **D341 HA1** | 7.26 | 0.31 | 0.20 | 19.82 | 6.26 | 1.18 | 0.19 |
| **D341 HA2** | 1.51 | 0.46 | 0.39 | 17.51 | 5.77 | 0.79 | 0.86 |
| **D341 LA1** | 22.98 | 0.29 | 0.02 | 10.53 | 0.16 | 0.00 | 2.35 |
| **D341 LA2** | 20.20 | 0.44 | 0.01 | 14.20 | 0.81 | 0.00 | 0.07 |
| **D347 HA1** | 2.61 | 1.06 | 0.28 | 26.27 | 11.55 | 0.61 | 0.06 |
| **D347 HA2** | 1.89 | 0.41 | 0.08 | 26.16 | 2.48 | 0.39 | 0.09 |
| **D347 LA1** | 32.27 | 0.20 | 0.02 | 16.43 | 0.06 | 0.00 | 0.35 |
| **D347 LA2** | 15.18 | 0.63 | 0.02 | 12.04 | 0.71 | 0.00 | 0.04 |

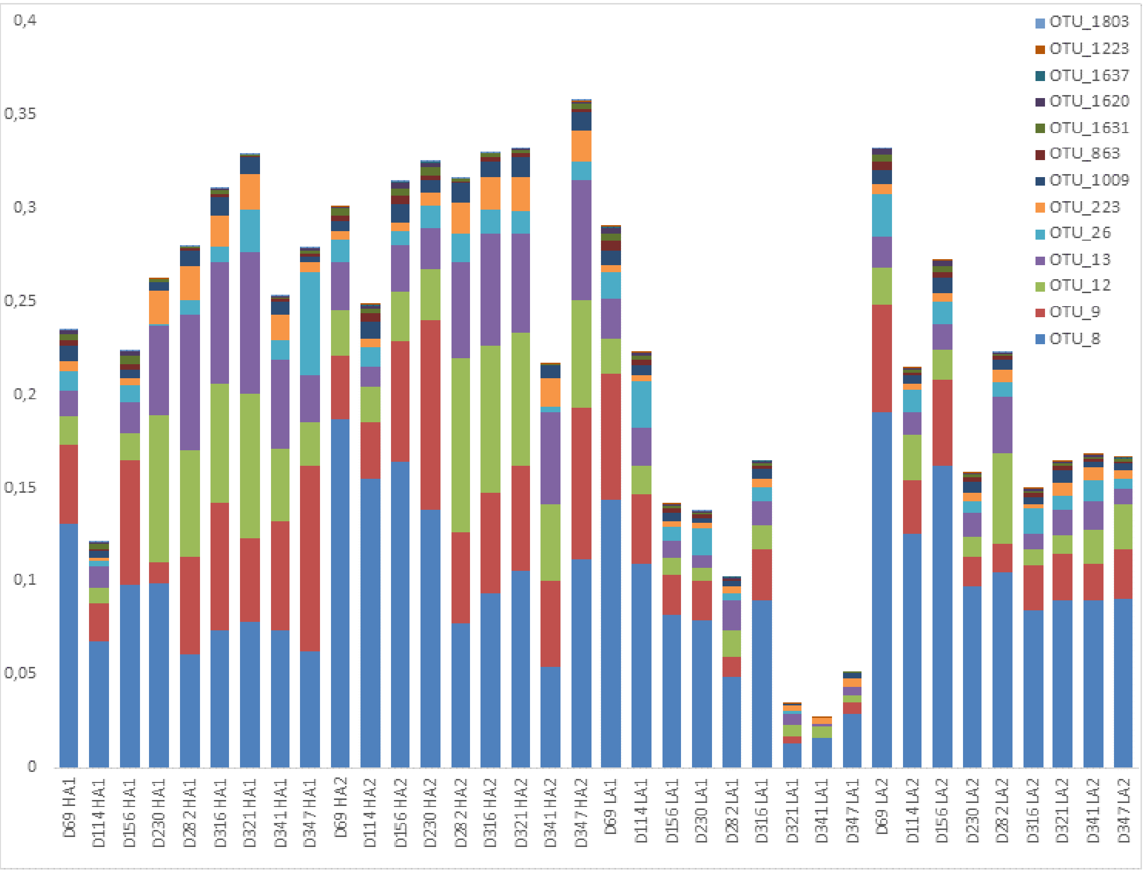


**Figure S1**. Relative abundances of 13 non-methanogen archaeal OTUs (see Fig. 4) in the reactor communities. D indicates experimental day.

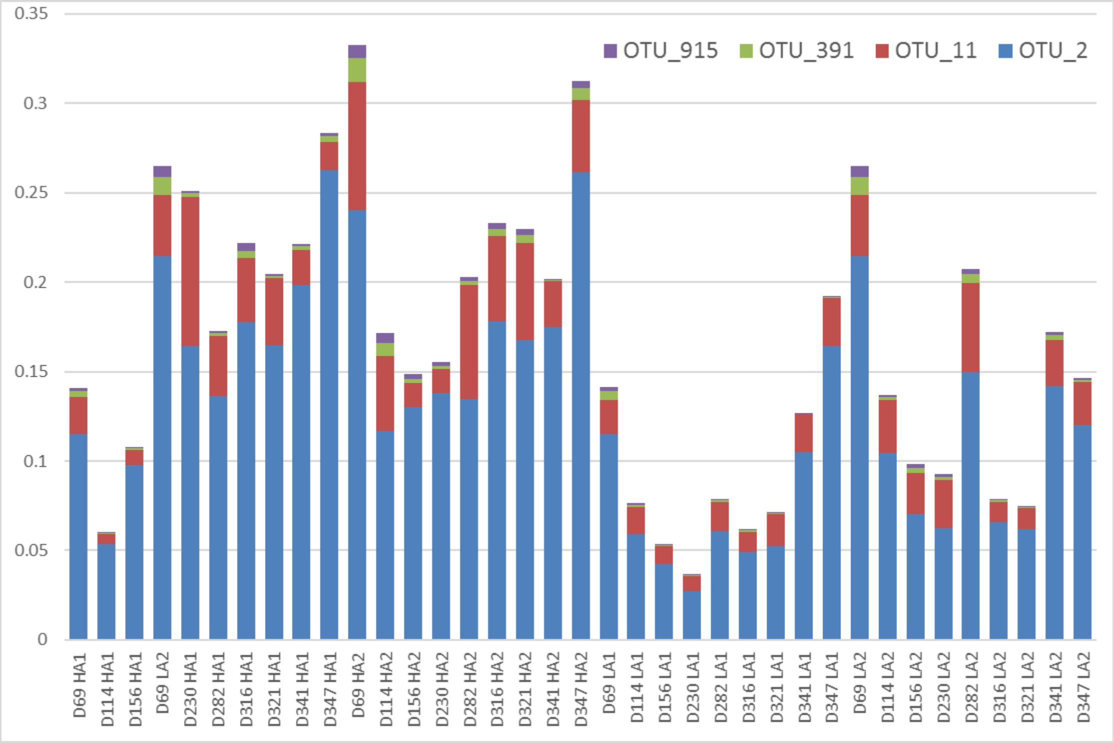


**Figure S2.** Relative abundance of the four most abundant *Methanosaeta* OTUs in the archaeal reactor communities. D indicate experimental day.

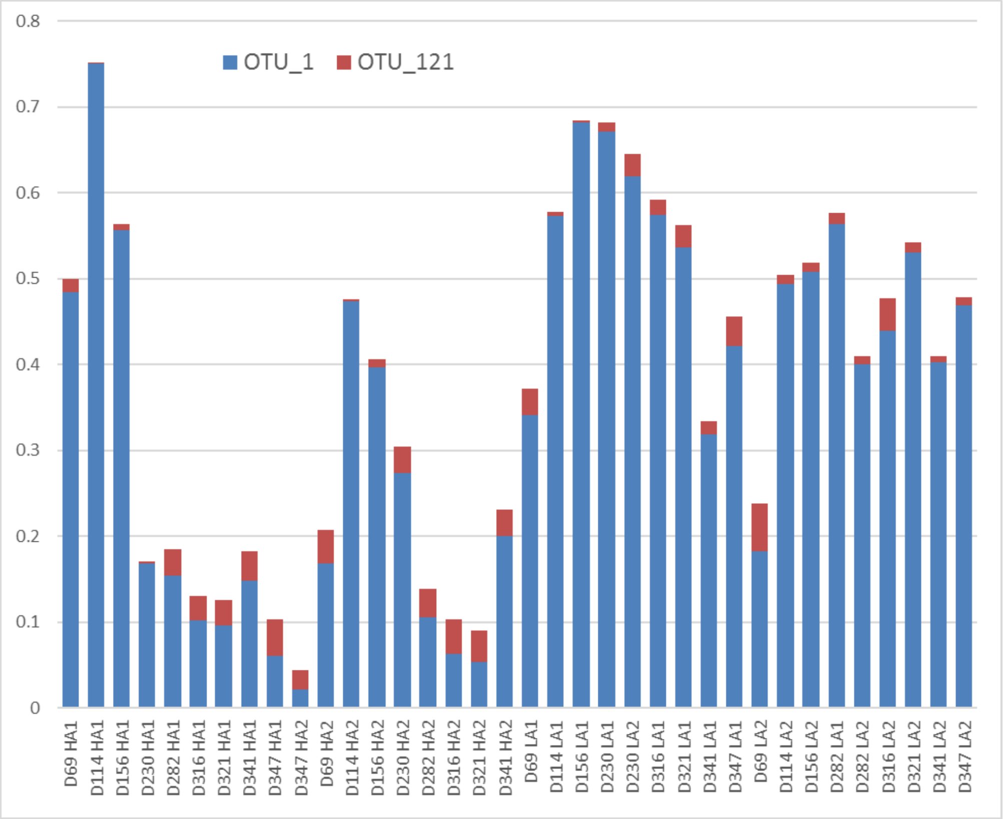


**Figure S3**. Relative abundance of the two most abundant *Methonosarcina* OTUs in the archaeal reactor communities. D indicate experimental day.

FAN was calculated from day 114 using equations S1 and S2.

$$K_a = \frac{[NH_3][H^+]}{[NH_4^+]} \tag{S1}$$

$$FAN = \frac{TAN}{1+\frac{10^{-pH}}{K_a}} \tag{S2}$$
